# Insulin‐like growth factor‐1 infusion in preterm piglets does not affect growth parameters of skeletal muscle or tendon tissue

**DOI:** 10.1113/EP092010

**Published:** 2024-07-09

**Authors:** Malene Tangbjerg, Ann Damgaard, Anders Karlsen, Rene B. Svensson, Peter Schjerling, Miriam Gelabert‐Rebato, Stanislava Pankratova, Per Torp Sangild, Michael Kjaer, Abigail L. Mackey

**Affiliations:** ^1^ Institute of Sports Medicine Copenhagen Department of Orthopaedic Surgery Copenhagen University Hospital – Bispebjerg and Frederiksberg Copenhagen Denmark; ^2^ Center for Healthy Aging Department of Clinical Medicine University of Copenhagen Copenhagen Denmark; ^3^ Xlab, Center for Healthy Aging, Department of Biomedical Sciences, Faculty of Health and Medical Sciences University of Copenhagen Copenhagen Denmark; ^4^ Research Institute of Biomedical and Health Sciences (IUIBS) University of Las Palmas de Gran Canaria, Las Palmas de Gran Canaria Canary Islands Spain; ^5^ Comparative Pediatrics and Nutrition, Faculty of Health and Medical Sciences University of Copenhagen Frederiksberg Denmark; ^6^ Department of Neonatology Rigshospitalet Copenhagen Denmark; ^7^ Department of Pediatrics Odense University Hospital Odense Denmark

**Keywords:** growth, IGF‐1, postnatal

## Abstract

Prematurity has physical consequences, such as lower birth weight, decreased muscle mass and increased risk of adult‐onset metabolic disease. Insulin‐like growth factor 1 (IGF‐1) has therapeutic potential to improve the growth and quality of muscle and tendon in premature births, and thus attenuate some of these sequalae. We investigated the effect of IGF‐1 on extensor carpi radialis muscle and biceps brachii tendon of preterm piglets. The preterm group consisted of 19‐day‐old preterm (10 days early) piglets, treated with either IGF‐1 or vehicle. Term controls consisted of groups of 9‐day‐old piglets (D9) and 19‐day‐old piglets (D19). Muscle samples were analysed by immunofluorescence to determine the cross‐sectional area (CSA) of muscle fibres, fibre type composition, satellite cell content and central nuclei‐containing fibres in the muscle. Tendon samples were analysed for CSA, collagen content and maturation, and vascularization. Gene expression of the tendon was measured by RT‐qPCR. Across all endpoints, we found no significant effect of IGF‐1 treatment on preterm piglets. Preterm piglets had smaller muscle fibre CSA compared to D9 and D19 control group. Satellite cell content was similar across all groups. For tendon, we found an effect of age on tendon CSA, and mRNA levels of COL1A1, tenomodulin and scleraxis. Immunoreactivity for elastin and CD31, and several markers of tendon maturation, were increased in D9 compared to the preterm piglets. Collagen content was similar across groups. IGF‐1 treatment of preterm‐born piglets does not influence the growth and maturation of skeletal muscle and tendon.

## Abstract (highlights)

- **What is the central question of this study?**
  Does infusion of insulin‐like growth factor‐1 in preterm piglets stimulate growth parameters of muscle and tendon?
- **What is the main finding and its importance?**
  IGF‐1 treatment of preterm born piglets does not influence growth and maturation of skeletal muscle and tendon. This adds to the current knowledge on the effect of IGF‐1 on muscle and tendon growth, and is relevant for future research in potential therapies for premature infants.

## 1 INTRODUCTION

Premature birth has been linked to multiple developmental issues. Premature infants and piglets are characterized by lower body weight, smaller muscle cross sectional area and shorter stature, even when reaching term equivalent age (Ahmad et al., 2010; Andersen et al., 2016; Johnson et al., 2012; Möllers et al., 2022). The impaired growth seems to persist throughout childhood when compared to born‐at‐term peers (Rowe et al., 2011).

During postnatal development, growth rates of musculoskeletal tissue are exceptionally high (Davis & Fiorotto, 2009; Rudar et al., 2019). Insulin‐like growth factor 1 (IGF‐1) induces hypertrophy of muscle fibres by increasing protein synthesis and decreasing protein degradation (Hakuno & Takahashi, 2018; Velloso, 2008). To sustain the growing muscle, satellite cells (SCs) must proliferate and differentiate to be incorporated as myonuclei in the existing muscle fibres (Rudar et al., 2019; Schiaffino & Mammucari, 2011). In the early postnatal weeks, SC content peaks relative to the total myonuclear pool and then drops progressively, reflecting their differentiation and integration into the existing muscle fibres (Mesires & Doumit, 2002; White et al., 2010).

Similar to muscle, the rapid growth of tendon structure and functional capacity takes place around the time of birth (Kalson et al., 2015; McBride et al., 1988). The developing tendon expresses scleraxis and tenomodulin, markers of early and late differentiation, respectively, as well as collagen type I, collagen type III, elastin, tenascin C and markers of vascular differentiation (Halper, 2014; Petersen et al., 2002; Takimoto et al., 2012). Types I and III collagen are major components of the connective tissue in both tendon and muscle (Kannus, 2000; Light & Champion, 1984). Collagen fibrillogenesis takes place from the embryonic stages through the postnatal period, and the process is initiated with the deposition of short, immature fibril intermediates. As the tissue matures, the fibril intermediates are replaced by longer small diameter collagen fibrils arranged in a parallel fashion (Connizzo et al., 2013).

Plasma IGF‐1 is decreased dramatically in preterm infants (average 10 ng/mL) compared to both term‐born infants and the levels in utero at corresponding ages (>50 ng/mL in gestation week 23–30) (Chen & Smith, 2007; Hellström, Ley, Hansen‐Pupp et al., 2016, 2016; Möllers et al., 2022). IGF‐1 plays a vital role in the physiological promotion of growth in early development, as it decreases protein degradation and promotes protein synthesis (Hakuno & Takahashi, 2018; Velloso, 2008). In adult human tendon tissue, IGF‐1 can be seen around the tendon fibroblasts, and IGF‐1 plays a pivotal role in the synthesis of extracellular matrix (Olesen et al., 2006). Deletion of the IGF‐1 receptor in tendon and muscle of mature mice has revealed a reduction in tendon dimensions, diminished cell proliferation in response to mechanical loading (Disser et al., 2019), and reduced bodyweight and size and total number of muscle fibres (Schiaffino & Mammucari, 2011).

As the musculoskeletal system is vital for physical activity and metabolism, finding possible interventions that can promote overall growth is important. However, muscle and tendon development in premature infants, as well as in newborns in general, remains an area that has received little attention, with a lack of long‐term data on the impact of preterm birth on musculoskeletal health (Singer et al., 2021).

The objective of this study was to investigate the influence of IGF‐1 infusion on the development of musculoskeletal tissue in piglets born 10 days prematurely. The main hypothesis for the study is that both muscle fibres and tendon in premature piglets will be positively influenced by the increased levels of IGF‐1 and mediate a catch up‐growth comparable to their age‐matched controls. In muscle and tendon tissue, this would be indicated by an increase in muscle fibre size, satellite cell content, and altered ECM composition (collagen, elastin content), vascularization, gene expression, and overall growth.

## 2 METHODS

### 2.1 Ethical approval

This animal study protocol was approved by the Danish Animal Experiments Inspectorate (license no.: 2020‐15‐0201‐00520), and all experiments were carried out according to guidelines from the European Communities Council Directive 2010/63/EU of the European Parliament. Procedures aimed to follow ARRIVE guidelines for animal experimentation (Percie du Sert et al., 2020). The health status of each piglet was evaluated twice daily, using validated scoring systems. For more detail see Rasmussen et al. (2023). The authors of this paper understand the ethical principles under which the journal operates and that their work complies with the animal ethics checklist.

### 2.2 Study design

**FIGURE 1:**
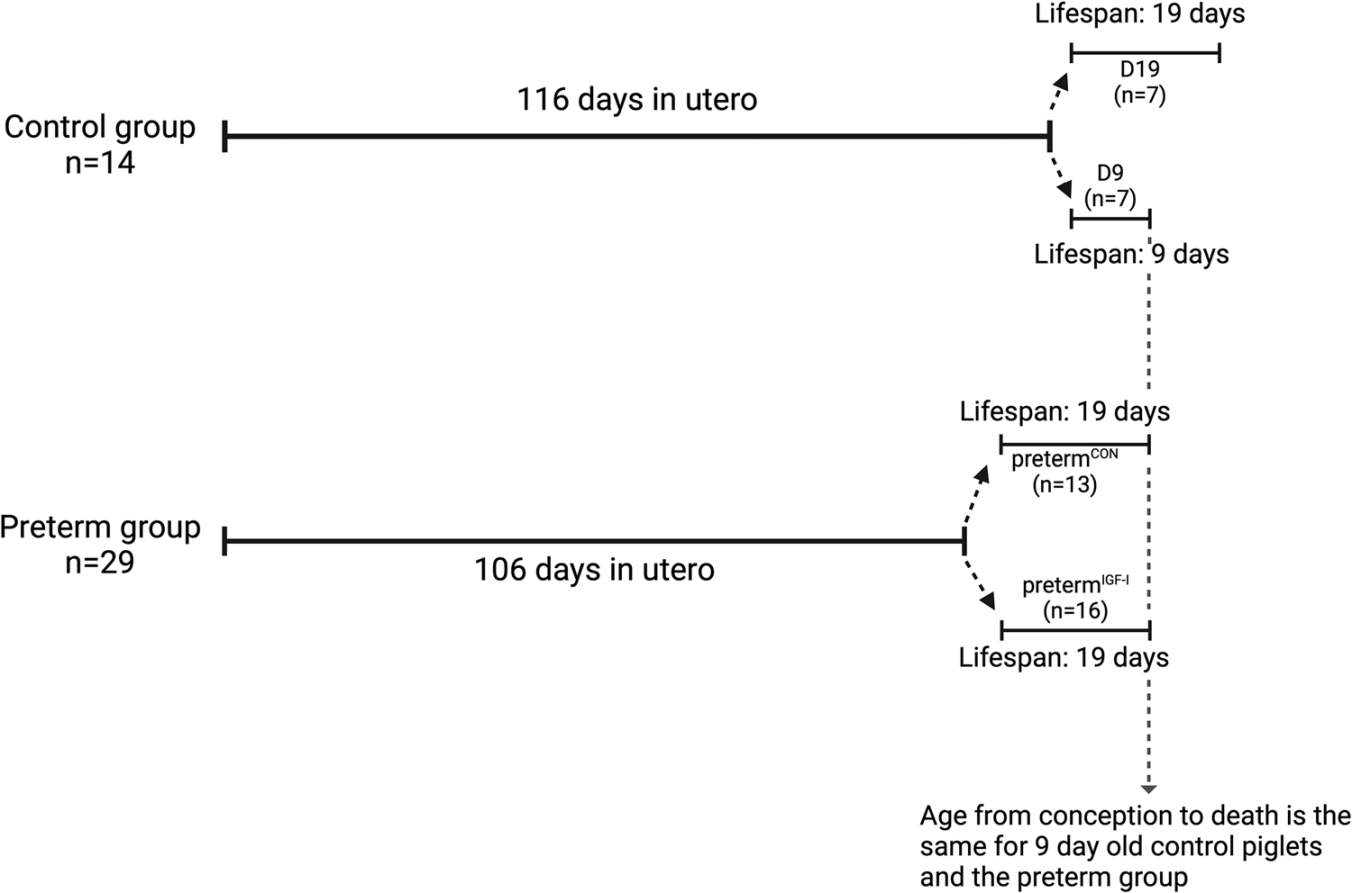
Overview of study design. Created with BioRender.com.

The design includes four groups of animals (Figure 1): preterm (born 10 days before term, reared for 19 days) IGF‐1 treated piglets (preterm^IGF‐1^, *n* = 16); a preterm control group (preterm^CON^, *n* = 13); a term‐born control group reared for 9 days (D9 term, *n* = 7); and a term‐born control group reared for 19 days (D19 term, *n* = 7). The D9 term piglets correspond to the same age since conception as the preterm piglets, while the D19 term piglets correspond to the same age since birth.

The preterm groups consisted of piglets (Landrace × Yorkshire × Duroc) from two litters that were born by caesarean section at gestational day 106 (90% of gestation, *n* = 29). The preterm piglets were stratified by sex and birth weight, and then randomized to either vehicle (*n* = 13 preterm^CON^), or IGF‐1 infusion (rhIGF‐1/binding protein‐3 (BP‐3), mecasermin rinfabate; *n* = 16 preterm^IGF‐1^).

Via intra‐arterial injection, the preterm^IGF‐1^ animals received a solution of 2.25 mg/kg/day IGF‐1 diluted in a formulation buffer on days 1–8. Meanwhile, the preterm^CON^ only received the formulation buffer (50 mM sodium acetate, 105 mM sodium chloride, 0.005% (v/v) polysorbate 20, pH 5.5, Takeda, Cambridge, MA, USA). From day 8 to 19, IGF‐1 or formulation buffer was administered via a subcutaneous catheter (Unomedical, Lejre, Denmark). The preterm^IGF‐1^ received an injection of 0.75 mL/kg three times daily, consistent with a total dose of 2.25 mg/kg/day. The preterm^CON^ received an injection of only the formulation buffer, in equal volume to the IGF‐1 injection, three times daily.

Feeding and housing conditions were identical across the two preterm groups, which were reared for 19 days. On days 1–10, the preterm piglets were housed in neonatal intensive care units and from day 10 to 19 they were transferred to bigger, individual cages. From day 1 to 7, the piglets received parenteral nutrition before being weaned and abruptly switched to enteral nutrition. The preterm piglets were weighed daily and the main growth outcomes have been published elsewhere (Rasmussen et al., 2023).

The term control piglets were from different litters than the preterm piglets. They were born vaginally and reared under farm conditions until sacrifice after either 9 or 19 days.

### 2.3 Plasma IGF‐1 levels

Circulating IGF‐1 levels were measured on postnatal day 18 in one litter of the piglets (preterm^IGF‐1^ *n* = 9, preterm^CON^ *n* = 8). The preterm piglets had blood drawn 1 h after an injection of IGF‐1/vehicle in the preterm piglets. As described previously (Rasmussen et al., 2023), IGF‐1 levels were quantified with a human IGF‐1 ELISA kit (Mediagnost GmbH, Reutlingen, Germany). The lower limit for detection was 20 ng/mL.

### 2.4 Growth

Overall growth of the preterm groups was assessed by comparing birth weight to their weight on the day of killing.

### 2.5 Tissue collection

Piglets were killed with intracardial sodium pentobarbital (euthanimal, ScanVet, Animal Health, Denmark). Within 40–60 min after killing, muscle and tendon tissue were surgically removed and samples of the extensor carpi radialis muscle and biceps brachii tendon were collected, embedded in TissueTek, frozen in isopentane pre‐cooled in liquid nitrogen and stored at −80°C.

### 2.6 Immunofluorescence, immunohistochemistry and microscopy

Using a cryostat at −20°C, 10 μm sections were cut and placed on microscope slides. From the tendon both cross‐sections and longitudinal sections were collected. Five piglets did not yield sufficient tendon tissue and were therefore excluded: one preterm^IGF‐1^ piglet and four preterm^CON^ piglets. From the muscle, cross‐sections were collected for all piglets. Sections were allowed to air dry and were then stored at −80°C until staining. An overview of the staining antibodies can be found in Supporting information. All sections were imaged on an Olympus BX51 microscope with an Olympus DP71 Camera using a 0.5× mount and a ×20 objective.

The SC‐71 monoclonal antibody developed by S. Schiaffino, the PAX7 monoclonal antibody developed by A. Kawakami and the 2E8 monoclonal antibody developed by E. S. Engvall were obtained from the Developmental Studies Hybridoma Bank, created by the NICHD of the NIH and maintained at The University of Iowa, Department of Biology, Iowa City, IA, USA.

### 2.7 Muscle fibre cross‐sectional area and fibre type distribution

The muscle sections were removed from the freezer and allowed to dry. Sections were fixed in 4% paraformaldehyde (PFA) for 5 min, then washed in Tris‐buffered saline (TBS) and incubated overnight in the fridge with a primary antibody against myosin heavy chain 2A (MyHC‐IIa). The following day, slides were washed and incubated with a secondary antibody and wheat germ agglutinin (WGA) for 60 min. After a final wash, sections were mounted using mounting medium containing 4′,6‐diamidino‐2‐phenylindole (DAPI).

Images were taken at different regions of the samples with no overlap (approximately six images, each covering an area of 0.57 mm^2^, from each sample, depending on size and section quality). Due to great variability in fibre type composition, the general MyHC‐IIa‐signal distribution in a sample was noted and then imaged at five to six locations scattered evenly to achieve representative images of the whole section. Imaging and the following analyses were performed by the same observer blinded to the treatment groups.

Images were analysed for muscle fibre CSA in a semi‐automatic manner using a modified version of a previously published macro (Karlsen et al., 2019) in ImageJ (version 1.53c; National Institutes of Health, Bethesda, MD, USA) to detect the WGA‐stained fibre outlines. For each sample, we aimed to include four images containing at least 200 fibres each. Five of the samples were only large enough for three images and two samples only had two images of sufficient quality. For these samples more fibres per image were included. On average, the total number of fibres included per sample was 1046 (SD = 187), ranging from 664 to 1478 fibres. From each sample, a mean CSA for MyHC‐IIa positive and MyHC‐IIa negative fibres was calculated separately. A mean CSA for all fibres regardless of fibre type was also determined.

For fibre type distribution, a threshold MyHC‐IIa intensity was determined manually for each sample and all fibres that were not automatically sorted as either clearly MyHC‐IIa‐positive or MyHC‐IIa‐negative were assessed by the observer or excluded (0–5 fibres per sample excluded). The data are reported as the mean percentage of MyHC‐IIa negative fibres per group.

Fibre type distribution patterns were assessed manually in the microscope at ×10 magnification. Regions where all fibres stained positively for MyHC‐IIa with an absence of ‘islets’ were denoted ‘A’, and regions with MyHC‐IIa negative fibres scattered among the MyHC‐IIa fibres as ‘islets’ were denoted as composition ‘B’ (Figure 3a).

As both patterns were often found within the same sample, a qualitative estimate of how much sample area exhibited the different kinds of fibre type distribution pattern was noted for each tissue sample. All samples were then categorized by their dominating fibre type distribution pattern, resulting in three categories: ‘mostly A’, ‘equal expression of A and B’ and ‘mostly B’ (denoted composition A, AB and B, respectively, see Figure 3b). Finally, the within‐group distribution of the three fibre type distribution patterns is reported as total number.

### 2.8 Muscle fibre central nuclei

The samples stained for MyHC‐IIa were manually screened for central nuclei in MyHC‐IIa positive and negative fibres. A central nucleus is defined as being free of the sarcolemma, although its position within the fibre may not necessarily be centralized. Values are expressed per 100 fibres. Results are reported as the median and range for each group.

### 2.9 Muscle satellite cells

The sections were washed in TBS followed by overnight incubation in the fridge with primary antibodies against Pax7 and Dystrophin. On the following day, the slides were washed in TBS and incubated for 60 min with secondary antibodies and WGA. They were then washed in TBS and incubated with Hoechst for 5 min. After a final wash in TBS, the sections were mounted using mounting medium without DAPI.

Images were taken at the same regions within each sample as the images obtained for CSA analysis on the previous section. Four to six images without overlap were taken of each sample with an average of 1400 fibres (SD = 110) included per sample. The criteria defining a satellite cell were positive signalling for Pax7, contrasted with the Hoechst–nuclei stain. All images were manually analysed in ImageJ at a standardized brightness and contrast level by the same observer, blinded to the treatment groups. The satellite cell data are expressed as number of satellite cells per 100 fibres for each sample.

### 2.10 Tendon CSA

CSA of the mid‐tendon on unstained cross‐sections was estimated in ImageJ by manually outlining the tendon. Cross‐sections were made after we had cut a few longitudinal sections, which underestimates the CSA slightly.

### 2.11 Tendon immunofluorescence

Two consecutive sections of tendon were stained for evaluation of the content of vessels (CD31, laminin), cells (DAPI) and elastin. The longitudinal biceps brachii tendon sections were removed from the freezer and allowed to dry. The first section was incubated overnight with primary antibodies against CD31 and laminin, and the second section was incubated overnight with primary antibodies against elastin and laminin. The next day sections were washed in TBS and incubated with secondary antibodies for 60 min. Sections were then washed in TBS, mounted in mounting medium containing DAPI, and dried in the dark for 2 days before imaging. The whole sample was imaged as two to eight overlapping images that were merged afterward (Preibisch et al., 2009). Imaging and analyses were performed by one blinded investigator using ImageJ software and a custom macro plugin designed for the specific staining.

The percentage area of DAPI and percentage area of laminin staining were evaluated in both the CD31/laminin and elastin/laminin images, and the mean value across the two protocols for each piglet was used for statistical analyses. Counting the number of nuclei was not possible in these images due to the significant clustering of cells. CD31 and elastin content were determined as the relative area of the sample stained positive.

### 2.12 Tendon collagen distribution

Collagen was stained using the picrosirius red (PSR) method. The sections were removed from the freezer and allowed to dry before they were fixed in Bouin's fluid (70% saturated aqueous picric acid, 5% acetic acid, 25% formalin) for 30 min, rinsed in distilled water and stained with 0.15% Fast Blue (F0500; Sigma‐Aldrich) in a magnesium borate buffer (7 mM magnesium sulphate, 28 mM sodium metaborate) for 10 min to enhance the contrast. After rinsing in distilled water, the sections were stained with 0.1% Direct Red 80 (catalogue number: 365548; Sigma‐Aldrich St. Louis, Missouri, United States) in saturated aqueous picric acid for 60 min. Finally, sections were rinsed in acidified water (0.5% glacial acetic acid) and picric alcohol (20% absolute ethanol, 10% saturated picric acid), dehydrated in 95% ethanol for 30 min followed by two rinses in 100% ethanol and one rinse in xylene before mounting with Pertex.

Image analysis consisted of dividing the PSR stain regions into four categories based on their hue, saturation and brightness on the hue–saturation–brightness spectrum: dense fully matured collagen (dark red areas), low density collagen (pink areas), immature collagen (orange areas, corresponding to mixed collagenous and non‐collagenous compounds, mainly cells) and non‐collagen (yellow). Each staining category is expressed as the percentage of tissue area after subtracting void areas. The literature shows that mature collagen stains red (Zhang et al., 2023), and we made the assumption that orange and pink is a continuum toward red mature collagen.

### 2.13 Tendon gene expression

#### 2.13.1 RNA extraction

Approximately fifty 20 μm‐thick cryosections from the embedded tendon tissue were homogenized in 1 mL of TriReagent (Molecular Research Center, Cincinnati, OH, USA) containing five stainless steel balls of 2.3 mm in diameter (BioSpec Products, Bartlesville, OK, USA) by shaking in a FastPrep‐24 instrument (MP Biomedicals, Illkirch, France) at speed level 4 for 15 s. Following homogenization, 100 μL bromo‐chloropropane was added to separate the samples into an aqueous and an organic phase. After isolation of the aqueous phase, RNA was precipitated using isopropanol. The RNA pellet was then washed in ethanol and subsequently dissolved in 20 μL RNase‐free water. Total RNA concentration and purity was determined by UV spectroscopy.

#### 2.13.2 RT‐qPCR

A total of 300 ng total RNA was converted into cDNA in 20 μL using the qScript cDNA Supermix (95048, Quantabio, Beverly, MA, USA) according to the manufacturer's protocol. For each target mRNA, 0.25 μL cDNA was amplified in a 25 μL SYBR Green polymerase chain reaction (PCR) containing 1 × QuantiTect SYBR Green Master Mix (Qiagen) and 100 nM of each primer. An overview of the primers used is given in Supporting information. The amplification was monitored in real time using the CFX96 Real‐time PCR machine (Bio‐Rad, Hercules, CA, USA). The *C*_t_ values were related to a standard curve made with known concentrations of DNA oligonucleotides (Ultramer™ oligos, Integrated DNA Technologies, Leuven, Belgium) with a DNA sequence corresponding to the sequence of the expected PCR product. The specificity of the PCR products was confirmed by melting curve analysis after amplification. RPLP0 mRNA was chosen as internal control. Data were log transformed before statistical analysis and presented as the geometric mean with back‐transformed SD in corresponding figures. Data are expressed relative to the geometric mean of the preterm^CON^ group.

#### 2.13.3 Statistics

All data were analysed using SigmaPlot 13.0 for Windows (Systat Software, San Jose, CA, USA) and GraphPad Prism (v.9.5.0, GraphPad Software, Boston, MA, USA). Data are presented as means with SD unless stated otherwise. Student's unpaired *t*‐test was used to compare the preterm^IGF‐1^ and preterm^CON^ for tendon mRNA data and all muscle data, except for central nuclei. The Mann–Whitney rank sum test was used for central nuclei data and the rest of the tendon data. Where the test between preterm^IGF‐1^ and preterm^CON^ groups showed no difference, the groups were pooled (referred to as preterm^COMBINED^) for further statistical analyses against the term control groups. For comparison of age groups, one‐way ANOVAs were carried out for muscle fibre CSA on both MyHC‐IIa positive and MyHC‐IIa negative fibres, and for fibre type distribution and SC content, as well as tendon mRNA, and followed by a *post hoc* Holm–Šidák test if the overall model was significant.

One‐way ANOVA on ranks (Kruskal–Wallis) was carried out for analyses of central nuclei, elastin, CD31, nuclei and laminin area percentage as well as the PSR staining, followed by Dunn's method for *post hoc* testing if the overall model was significant. The distribution of fibre type patterns (see Section 3) was analysed by Fisher's exact test. If significant, *post hoc* testing was conducted by applying Fisher's exact test to each group/pattern combination against all other combinations with Bonferroni correction for multiple comparisons.

*P* < 0.05 was considered statistically significant. Significant *P*‐values were reported as follows: *P < 0.05, **P < 0.01, ***P < 0.001, **** *P *< 0.0001.

## 3 RESULTS

### 3.1 Growth

Unpaired *t*‐tests revealed no statistically significant difference in final body weight (*P* = 0.13) or weight gain (*P* = 0.25) between preterm^IGF‐1^(1.8 kg, SD = 3.1 kg, *n* = 15) and preterm^CON^ (2.0 kg, SD = 0.3 kg, *n* = 9). Preterm^COMBINED^ had significantly lower final body weight (1.9 kg, SD = 0.3 kg) compared to D9 (3.2 kg, SD = 0.6 kg, *n* = 7) (*P *< 0.0001) and D19 (4.5 kg, SD = 1.1 kg, *n* = 7) (*P *< 0.0001). Further, D9 had significantly lower final body weight compared to D19 (*P* = 0.002).

### 3.2 Plasma IGF‐1 levels

Mean IGF‐1 levels were significantly higher (*P *< 0.0001) in preterm^IGF‐1^ (139 ng/mL, SD = 28, *n* = 9) compared to preterm^CON^ (43 ng/mL, SD = 7, *n* = 8), D9 (47 ng/mL, SD = 18, *n* = 7) and D19 (46 ng/mL, SD = 19, *n* = 6). There were no significant differences between preterm^CON^, D9 and D19 (*P* = 0.99).

### 3.3 Muscle fibre type distribution and cross‐sectional area

**FIGURE 2:**
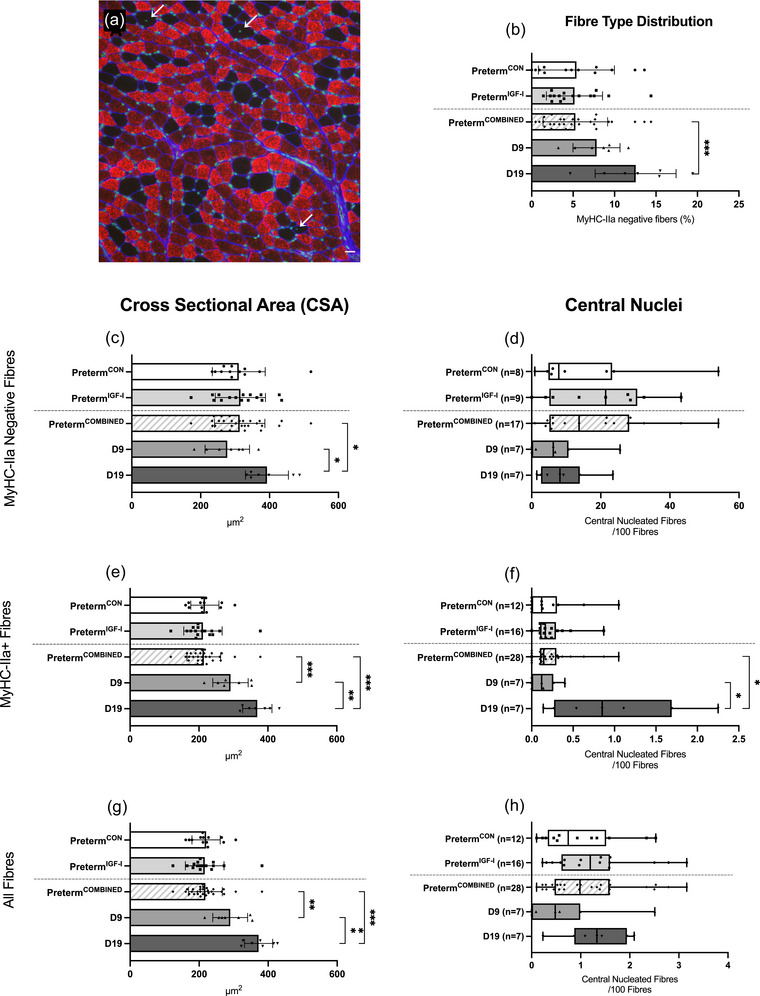
Muscle fibre cross‐sectional area, central nuclei and fibre typing. (a) Immunofluorescence image showing the low proportion of MyHC‐IIa negative fibres in a sample from a pig in the D19 group, compared to MyHC‐IIa positive fibres (red). Nuclei were stained with DAPI (green), and central nuclei are marked (arrows). Scale bar: 20 μm. (b) Percentage of MyHC‐IIa negative fibres in each group. Data are means ± SD. ***P < 0.001. (c, e, g) Cross‐sectional area of muscle fibres overall (g) and by type (c, e) in each group. Data are means ± SD. ***P < 0.001, **P < 0.01, *P < 0.05. (d, f, h) Central nuclei content in each group irrespective of fibre type (h) and by type (d, f). Data are medians with range and all individual sample values plotted. *P < 0.05.

Overall, all samples consisted predominantly of MyHC‐IIa fibres (Figure 2a). In the preterm groups, muscle consisted of approximately 5% MyHC‐IIa negative fibres, with no significant difference between preterm^IGF‐1^ (5.1%, SD = 3.4, *n* = 16) and preterm^CON^ (5.4%, SD = 4.6, *n* = 13) (*P* = 0.835). We found a significantly higher percentage of MyHC‐IIa negative fibres in the D19 (12.5%, SD = 4.9, *n* = 7) compared to the preterm group (*P *< 0.001), while the D9 group (7.8%, SD = 2.8, *n* = 7) had values between the preterm and D19 but was not significantly different from either (*P* = 0.15 and *P* = 0.051, respectively; Figure 2b).

There was no effect of IGF‐I on muscle fibre CSA for either MyHC‐IIa or MyHC‐IIa negative fibres, and these data were therefore combined before being compared with D9 and D19. D9 had significantly larger MyHC‐IIa positive fibres compared to preterm^COMBINED^. D19 had significantly larger MyHC‐IIa positive and MyHC‐IIa negative fibres compared to D9 and preterm^COMBINED^ (Figure 2c,e). Due to the overall low prevalence of MyHC‐IIa negative fibres, the difference in CSA for all fibres combined resembled the CSA data for MyHC‐IIa fibres (Figure 2e,g).

**FIGURE 3:**
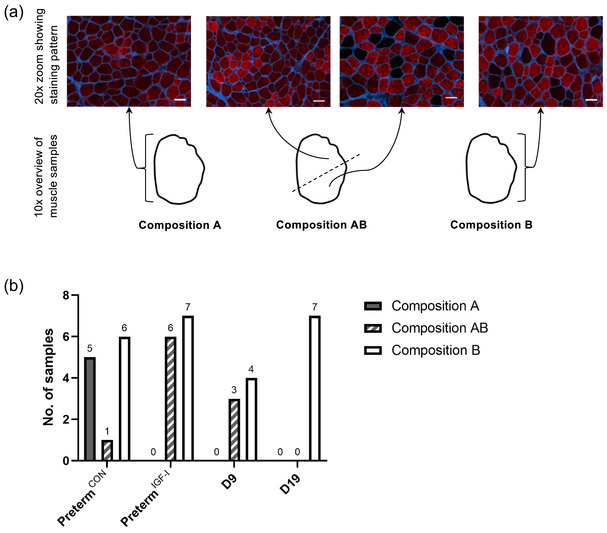
Muscle fibre type distribution patterns. (a) Immunofluorescence images stained for MyHC‐IIa (red) showing two different regions within the same muscle sample. Regions with scattered distinctly negative fibres (composition B) exist alongside regions with varying MyHC‐IIa signal intensity but no clearly negative fibres (composition A). Scale bars: 20 μm. Alongside is a schematic representation of three muscle samples to illustrate how each sample was characterized by its dominant staining pattern: composition B is dominated by ‘islets’; composition A is dominated by the absence of ‘islets’; and composition AB has an equal distribution of areas with and without ‘islets’. (b) Bar plot showing the within‐group distribution of staining patterns.

We found two distinct patterns of fibre type distribution (see Section 2 and Figure 3a), typically simultaneously present within the same sample, though the composition varied greatly between samples. The preterm^CON^ group contained samples of all three composition types (‘A’, ‘B’ and ‘AB’), while neither the preterm^IGF‐1^, D9 nor D19 group contained any samples dominated by composition ‘A’ (Figure 3b). In the D19 group, all samples were characterized by the ‘islet’‐pattern (‘B’). We found a significant difference in the distribution across the groups (*P* = 0.038), but *post hoc* testing was unable to identify any specific group/pattern combination as significantly different.

### 3.4 Muscle fibre central nuclei

Central nuclei‐containing fibres were found in all samples except one, although in very small numbers (Figure 2d,f,h). Several of the preterm samples (5 preterm^CON^, 7 preterm^IGF‐1^) were excluded from the data on MyHC‐IIa negative central nuclei as they contained too few MyHC‐IIa negative fibres. The median numbers of MyHC‐IIa negative fibres in the excluded samples were nine (range = 0–18) for preterm^CON^ and 29 (range = 34–13) for preterm^IGF‐1^, whereas the medians in the included samples were 68 (range = 146–37, *n* = 8) and 65 (range = 173–37, *n* = 9), respectively.

There was no significant difference in the median percentage of fibres with central nuclei between groups across all fibres, nor was there any significant difference within the MyHC‐IIa negative fibres. However, for MyHC‐IIa positive fibres there was a significantly higher median percentage of centrally nucleated fibres in D19 (0.85%, range = 2.25–0.14, *n* = 7) than D9 (0.12%, range = 0.40–0.00, *n* = 7) (*P* = 0.012) and the preterm^COMBINED^ (0.15%, range 0–1.05, *n* = 28) (*P* = 0.012) (Figure 2f).

### 3.5 Muscle satellite cells

**FIGURE 4:**
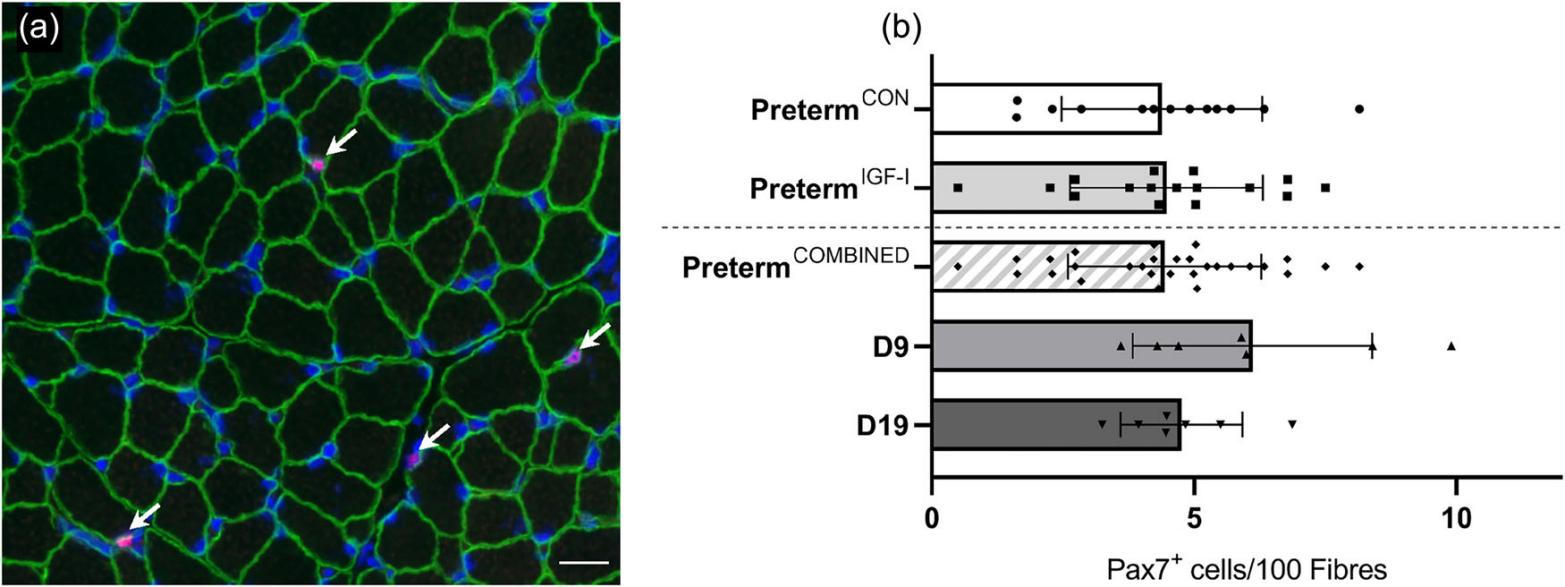
Muscle fibre satellite cells. (a) Immunofluorescence image with arrows marking satellite cells (pink, Pax7^+^), characterized by their position adjacent to the sarcolemma (green, dystrophin) and control‐stained with Hoechst (blue, marks all nuclei). Scale bar: 20 μm. (b) Percentage of satellite cells in the muscle samples from each group. Data are means ± SD. Preterm^CON^ *n* = 13, preterm^IGF‐1^ *n* = 16, D9 *n* = 7, D19 *n* = 7.

Satellite cell content was similar in all groups (preterm^CON^ (*n* = 13) and preterm^IGF‐1^ (*n* = 16), *P* = 0.897; D9 (*n* = 7), D19 (*n* = 7) and preterm^COMBINED^ (*n* = 29), *P* = 0.109; Figure 4b).

### 3.6 Tendon CSA

Tendon CSA did not differ between preterm^IGF‐1^ (1.74 mm^2^, SD = 0.72, *n* = 14) and preterm^CON^ (1.72 mm^2^, SD = 0.45, *n* = 9) (*P* = 0.938). One preterm^IGF‐1^ tendon was excluded from tendon CSA analysis due to poor sectioning of the sample. The CSA in preterm^COMBINED^ was 1.73 mm^2^ (SD = 0.62, *n* = 23). The CSA of D9 was 2.76 mm^2^ (SD = 0.67, *n* = 7), which was not significantly larger than the preterm group (*P* = 0.070). Further, the CSA of D19 was 4.55 mm^2^ (SD = 1.88, *n* = 7) and thus significantly larger than preterm^COMBINED^ (*P *< 0.0001) and D9 (*P* = 0.008).

### 3.7 Tendon vascularization

Preterm^IGF‐1^ and preterm^CON^ had an equal proportion of tendon tissue stained positive for CD31 (2.82%, SD = 0.01%, *n* = 14 and 2.85%, SD = 0.01%, *n* = 9, respectively; *P* = 0.78) and laminin (7.02%, SD = 0.02%, *n* = 14 and 6.25%, SD = 0.01%, *n* = 8, respectively; *P* = 0.21). One preterm^IGF‐1^ sample was excluded from all statistical analysis as it contained no tendon tissue and one preterm^CON^ sample was further excluded from statistical analysis of mean laminin and nuclei percentage due to lack of tendon tissue.

**FIGURE 5:**
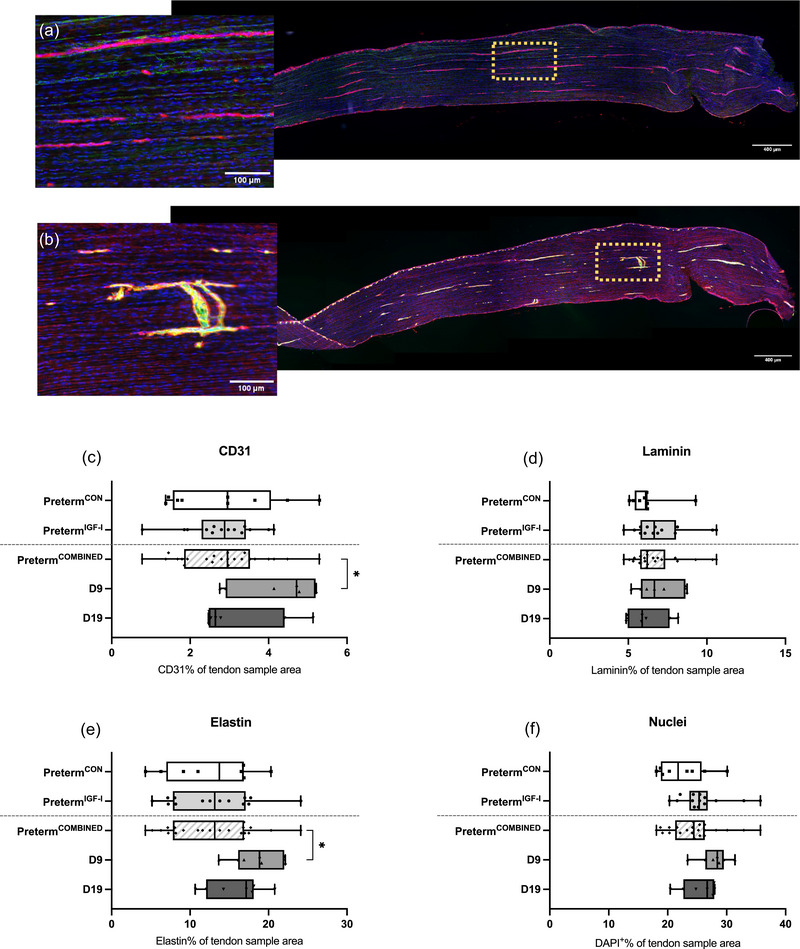
Tendon histology (immunofluorescence staining). (a) Immunofluorescence staining of tendon section, elastin (red), laminin (green) and nuclei (blue). (b) Immunofluorescence staining of tendon section, CD31 (red), laminin (green) and nuclei (blue). (c–f) Results depicted as the percentage of the whole sample area, reported as medians with range and individual sample values plotted. *P < 0.05. (c) Preterm^CON^ *n* = 9, Preterm^IGF‐1^ *n* = 14, D9 *n* = 7, D19 *n* = 7. (d–f) Preterm^CON^ *n* = 8, Preterm^IGF‐1^ *n* = 14, D9 *n* = 7, D19 *n* = 7.

We found a significantly greater CD31 content in D9 (4.25%, SD = 0.01%, *n* = 7) compared to preterm^COMBINED^ (2.82%, SD = 0.01%, *n* = 23) (*P* = 0.04). There was no significant difference between D19 (3.21%, SD = 0.01%, *n* = 7) and preterm^COMBINED^ (*P* = 1.00), and between D9 and D19 (*P* = 0.21) (Figure 5c). Laminin was similar between all age groups (*P* = 0.54).

### 3.8 Tendon elastin

Preterm^IGF‐1^ and preterm^CON^ had an equal percentage of tendon tissue stained positive for elastic fibres (12.9%, SD = 0.05%, *n* = 14 and 12.7%, SD = 0.06%, *n* = 8, respectively) (*P* = 0.865). Again, we found an effect of age group (*P* = 0.029) that was attributed to significantly greater elastin content in D9 (18.4%, SD = 0.03%, *n* = 7) than preterm^COMBINED^ (*P* = 0.036). When comparing D19 (15.7%, SD = 0.04%, *n* = 7) with the preterm^COMBINED^ (12.7%, SD = 0.05%, *n* = 22) (*P* = 0.418) and D9 (*P* = 1.000) we found no significant differences (Figure 5e).

### 3.9 Tendon nuclei

There were no differences in the percentage of area covered by nuclei in the preterm^IGF‐1^ (25.9%, SD = 0.04%, *n* = 14) and preterm^CON^ (22.5%, SD = 0.04%, *n* = 8) (*P* = 0.061), nor was there any significant effect of age between preterm^COMBINED^ (24.7%, SD = 0.04%, *n* = 22), D9 (27.9%, SD = 0.03%, *n* = 7) and D19 (25.5%, SD = 0.03%, *n* = 7) (*P* = 0.052) (Figure 5f).

### 3.10 Tendon collagen distribution

**FIGURE 6:**
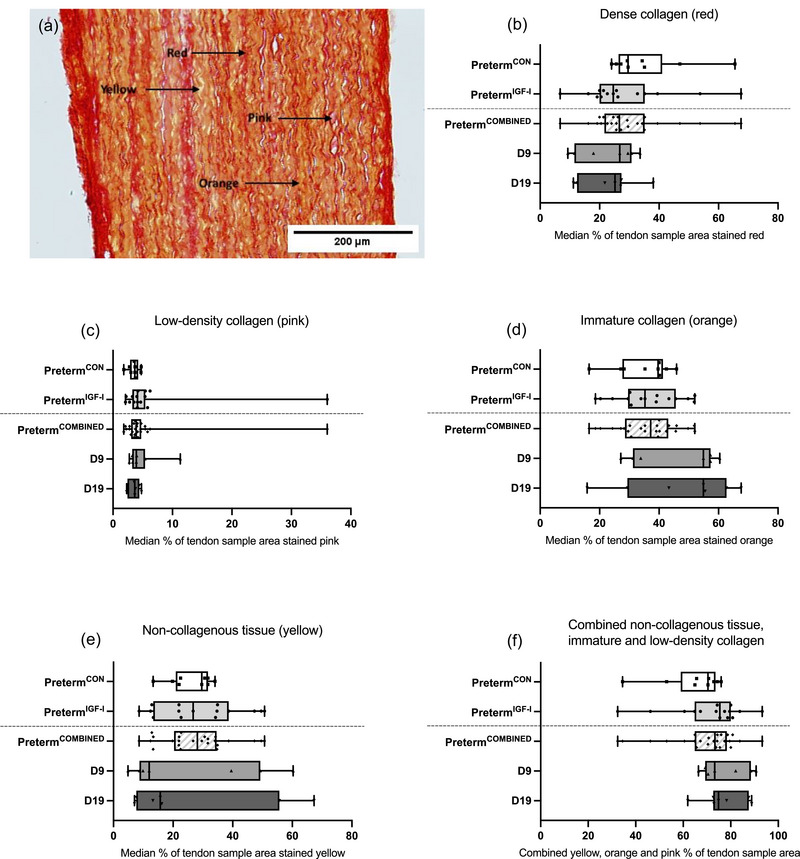
Tendon histology (PSR). (a) Zoomed in image of picrosirius red staining. (b–f) Results of the picrosirius red staining showing percentage of tendon sample area stained red (dense fully matured collagen), pink (low density collagen), orange (mixed collagenous and non‐collagenous compounds), yellow (non‐collagen). Data are medians with range and individual sample values plotted. Preterm^CON^ *n* = 9, Preterm^IGF‐1^ *n* = 15, D9 *n* = 7, D19 *n* = 7.

There were no significant differences between preterm^IGF‐1^ (*n* = 15) and preterm^CON^ (*n* = 9) in the relative extent of dense collagen (red, Figure 6b) in tendons from preterm piglets (*P* = 0.121), or the low‐density collagen (pink, Figure 6c, *P* = 0.170), immature (orange, Figure 6d, *P* = 0.905) or non‐collagenous (yellow, Figure 6e, *P* = 0.633) areas, or in the pink, orange and yellow areas combined (Figure 6f, *P* = 0.095). When comparing the three age groups, there were no differences in any of the collagen staining types: red (*P* = 0.379), yellow (*P* = 0.532), orange (*P* = 0.099) and pink (*P* = 0.738) or the areas combined (*P* = 0.406) (Figure 6b, d, f). A representative image of the PSR staining can be seen in Figure 6a.

### 3.11 Tendon gene expression

**FIGURE 7:**
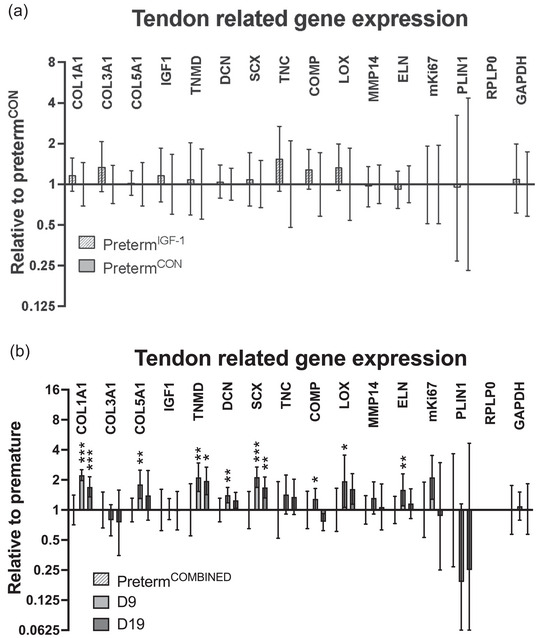
Tendon related mRNA expression. (a) Tendon related mRNA expression in preterm^IGF‐1^ and preterm^CON^. (b) Tendon related mRNA expression in preterm^COMBINED^, D9 and D19 groups. Collagen type I (*COL1A1*), collagen type II (*COL1A3*), collagen type V (*COL5A1*), insulin‐like growth factor 1 (*IGF1*), tenomodulin (*TNMD*), decorin (*DCN*), scleraxis (*SCX*), tenascin C (*TNC*), cartilage oligomeric matrix protein (*COMP*), lysyl oxidase (*LOX*), matrix metallopeptidase 14 (*MMP14*), elastin (*ELN*), perilipin 1 (*PLIN1*), ribosomal protein lateral stalk subunit P0 (*RPLP0*), glyceraldehyd‐3‐phosphate dehydrogenase (*GAPDH*). Preterm^IGF‐1^ *n* = 15, preterm^CON^ *n* = 9, D9 *n* = 7, D19 *n* = 7.

Comparing preterm^IGF‐1^ (*n* = 15) and preterm^CON^ (*n* = 9), no significant differences were found in mRNA levels of markers of tendon tissue development (*IGF‐1*, *TNMD*, *TNC*, *COL3A1*, *ELN*) as well as collagen fibrillogenesis and maturation (*COL1A1*, *COL5A1*, *MMP14*, *SCX*, *DCN*, *LOX*; Figure 7a).

Compared to the preterm^COMBINED^ there was a significantly higher expression of the following genes in D9 (*n* = 7) and D19 (*n* = 7): *COL1A1* (*P *< 0.001 in both groups), *TNMD* (*P* = 0.006 in D9 and *P* = 0.011 in D19) and *SCX* (*P *< 0.001 in D9 and *P* = 0.005 in D19) (Figure 7b).

D9 had significantly higher expression of *DCN* (*P* = 0.007), *COL5A1* (*P* = 0.001), *LOX* (*P* = 0.011) and *ELN* (*P* = 0.007) compared to preterm^COMBINED^. There were no significant differences in these genes when comparing preterm^COMBINED^ and D19, or when comparing D9 and D19.

D9 had significantly higher expression of *COMP* (*P* = 0.036) compared to D19, with no significant differences between D19 and preterm^COMBINED^ or D9 and preterm^COMBINED^. There was no significant effect of group on *COL3A1* (*P* = 0.284), *IGF‐1* (*P* = 0.984), *PLIN1* (*P* = 0.051), *TNC* (*P* = 0.257) or *MMP14* (*P* = 0.238).

## 4 DISCUSSION

### 4.1 Effect of IGF‐1

The present study investigated muscle and tendon histology in a preterm piglet model receiving IGF‐1 infusion. We found no significant impact of 19 days of IGF‐1 treatment on the growth and development of muscle and tendon, whereas our secondary analyses revealed significant effects of age and prematurity per se.

Our findings regarding the effect of IGF‐1 supplementation are consistent with a study conducted on premature infants, where the administration of IGF‐1 through parenteral nutrition failed to demonstrate any influence on growth indicators such as weight and size of biceps and triceps muscle (Corpeleijn et al., 2008). Similarly, a study on mature piglets revealed that supplementing their milk formula with IGF‐1 did not affect the growth of the internal organs (Xu et al., 1994).

There is a paucity of studies investigating muscle growth in response to IGF‐1 infusion, and papers with a similar design to the present study have conflicting conclusions on the hypertrophic effect of IGF‐1. One study in adolescent rats found increased CSA of type I, type IIa and type IIx diaphragmatic fibres in response to IGF‐1 (Lewis et al., 1997), while a poultry model reported no effect on growth rate and even a significant decrease in skeletal muscle weight in response to IGF‐1 infusion (Czerwinski et al., 1998). It should be noted that our findings on preterm skeletal muscle in this study are based on a muscle dominated by type II muscle fibres (approximately 95%), so we cannot rule out a potential effect of IGF‐1 on type I muscle fibres.

Myofibres displaying a central nucleus are believed to be newly formed (Cadot et al., 2015). To assess the potential impact of IGF‐1 on muscle hyperplasia, we measured the presence of centrally nucleated fibres. The results suggest that there were only a few such fibres present in the samples and no significant differences could be detected between the preterm^IGF‐1^ and preterm^CON^ groups. Consequently, our findings do not support any hyperplastic effect of IGF‐I treatment, and due to the low prevalence of centrally nucleated fibres, we are confident that hyperplasia had no effect on our overall conclusion from the CSA analysis.

We also investigated the satellite cell pool size to determine whether IGF‐I influenced satellite cell proliferation and found no significant difference between the preterm^IGF‐I^ group and the control group. This is in concordance with the overall conclusions of the CSA analysis, and the lack of hyperplasia. In support of our findings, another porcine study on isolated, cultured satellite cells from piglets with low and normal birth weight reported that stimulation with IGF‐1 had no effect on proliferation or differentiation of satellite cells into myotubes (Chen et al., 2017).

Contrary to our initial hypothesis, we failed to detect any influence of IGF‐1 supplementation on tendon collagen in the preterm piglets, despite the increased level of circulating IGF‐1 in preterm^IGF‐1^ compared to preterm^CON^ (Rasmussen et al., 2023). This lack of influence was evident in both the mRNA expression levels and the histological analyses. Previous studies on in vitro tendon constructs have shown that IGF‐1 supplementation increases fibril diameter along with elevated mRNA expression levels of collagen type I and III, tenomodulin and scleraxis (Herchenhan et al., 2015). Moreover, Doessing et al. reported from human studies that increasing IGF‐1 levels through recombinant human growth hormone (rhGH) injections led to enhanced fractional synthesis rates of collagen in the muscle and tendon of healthy adults (Doessing et al., 2010).

It is plausible that during the postnatal phase, preterm piglets may be unable to effectively utilize surplus IGF‐1 due to a possible plateau in growth rate, thereby limiting its potential impact on muscle and tendon growth.

### 4.2 Effect of prematurity

The D9 term and preterm piglets have the same age from conception to death, but the D9 group have a 10‐day longer in utero lifespan. While we found no effect of IGF‐1 on growth performance in preterm piglets, several of our secondary exploratory data point to a negative effect of prematurity on parameters of growth. In preterm tendon, this is evidenced by lower levels of postnatal tendon maturation markers compared to D9 piglets, and significantly smaller tendon CSA compared to D19. In muscle tissue, we found significantly smaller muscle fibres in preterm^COMBINED^ compared to D9 and D19 term piglets, indicating that they did not have sufficient catch‐up growth to reach a comparable muscle mass to the term equivalent D9 term group. It should be noted that the delivery mode, environment and feeding conditions were very different between the preterm and term groups, which might impact the comparison between the preterm and term groups. However, previous work by Andersen et al. (2016) show that preterm piglets, reared under the same conditions as term piglets, have reduced body growth, reduced muscle mass and reduced IGF‐1 levels.

An alternative explanation for the lack of response in muscle and tendon to IGF‐1 treatment could be that prematurity per se hinders normal protein synthesis. Recent studies suggest that prematurity blunts the normal anabolic stimulus of feeding, as the increase in protein synthesis after feeding is significantly lower in preterm subjects (Naberhuis et al., 2019; Rudar et al., 2021). One proposed explanation is that the full capacity for protein synthesis develops late in gestation. Most notably, the insulin signalling pathway is negatively affected by prematurity. A later study has shown that translation initiation was negatively affected by prematurity. In preterm piglets (103 days in utero), insulin‐induced 4EBP1 phosphorylation, and increased abundance of eIF4E·eIF4G, was attenuated by prematurity, concurrent with a 22% lower protein synthesis rate (*P *< 0.05) (Rudar et al., 2021). These findings are highly relevant to our study of IGF‐1 treatment, as the pathway for translation initiation is the same for insulin and IGF‐1. Consequently, IGF‐1 may not elicit an efficient hypertrophic response if the capacity for protein synthesis is inherently poorer in premature individuals because of their disrupted gestational development. On the other hand, it has been shown that treatment with IGF‐1 stimulates protein synthesis in the cerebellum of preterm piglets, suggesting a difference between musculotendinous tissue and other organ systems (Christiansen, Holmqvist et al., 2023; Christiansen, Ventura et al., 2023).

### 4.3 Effect of age

In muscle, we found that fibre size increased with maturation, as the mean MyHC‐IIa and MyHC‐IIa negative fibre CSA of D19 term piglets were significantly larger than for the D9 term piglets. The proportion of MyHC‐IIa negative fibres in extensor carpi radialis increased with age, likely reflecting maturation of the muscle. The finding of two distinct patterns of fibre type distribution and the great variance in their representation within and between samples might reflect the immaturity of the muscle in all groups. The characteristic ‘islet’‐pattern likely reflects a more mature muscle composition, as all muscle samples from the D19 group were characterized by this pattern. As the D9 and D19 groups did not contain any samples dominated by composition ‘A’, this pattern likely reflects a more immature muscle, while the mixed‐type composition (‘AB’) might reflect a transition state between the immature and mature muscle composition. The pattern of one or a few MyHC‐IIa negative fibres as an islet surrounded by type II fibres is similar to earlier findings and reflects the secondary myogenesis that takes place in porcine muscle (Bérard et al., 2011; Lefaucheur et al., 2002).

In comparison to more developed fetuses, less mature fetuses exhibit a higher level of collagen type III and lower levels of collagen type I in tendon (Macedo et al., 2022), and total collagen content increases with age (Ansorge et al., 2011). These differences are reflected in our data on expression of mRNA for *COL1A1*, where we observed significantly higher levels of tendon *COL1A1* mRNA in D9 and D19 term piglets when compared to preterm piglets. We did not see an effect of age on tendon *COL3A1* expression. An earlier study found that *COL5A1*, essential for the fibrillogenesis of collagen types I and III, decreased throughout the entire postnatal period (Wenstrup et al., 2011). We found *COL5A1* to be highest in the D9 term group. Since we do not have an earlier time point we cannot state whether we actually see a decrease over time aligning with Wenstrup et al., or whether the D9 value represents a peak.

Regarding the maturity of collagen at the protein level, it is well documented that type I collagen exhibits an orange‐red staining pattern when subjected to the PSR protocol (Junqueira et al., 1978). Building on previous data (Zhang et al., 2023), we argue that the observed colour variation can be attributed to the developmental stage of the tendons in these very young piglets. Data from both humans and mice found that collagen fibres become denser and align in a more parallel fashion, and that collagen content increases with increasing age (Fan et al., 2022; Jiang et al., 2021; Macedo et al., 2022). Our findings did, however, not reveal any distinctions in the distribution of red, pink and orange areas among the three age groups. This could be a reflection of tissue immaturity. A study on mice proposes that postnatal day 7–14 is the most important phase for postnatal tendon maturation (Fan et al., 2022). Our data show that tendon is growing and maturing in the same manner, regardless of the different age groups and despite the difference in CSA. The explanation for this could be that the piglets in this study are most likely all in the beginning of the most important postnatal tendon maturation phase, described previously (Fan et al., 2022), when taking into account the less rapid development in piglets compared to mice. The collagen might not begin to fully mature before they are past the postnatal period.

Previous studies examining tendon tissue in postnatal mice and rats have demonstrated a gradual reduction in cell content (Chen et al., 2016; Fan et al., 2022). This pattern has also been observed in human fetuses at gestation week 22–28 and 32–38 (Macedo et al., 2022). Our data demonstrated no significant difference in the relative tissue area occupied by DNA among the three groups, further supporting that the tendon remains in an immature state even in the D19 term piglets.

Around postnatal D9, previous studies (Kelleher et al., 2004; Zhang et al., 2006) have identified a pattern with a peak in multiple markers related to postnatal maturation and development of tendon. In our study, the D9 group displayed the highest level of elastin staining, as well as gene expression of *ELN*, *DCN* and *LOX*. It is important to point out, that since we do not have an earlier time point, and due to potential species differences in expression dynamics, we do not know whether our D9 findings correspond to the D9 peak values of the earlier studies (Kelleher et al., 2004; Zhang et al., 2006). Existing literature reports a steady decrease of blood vessels throughout the postnatal period (Meller et al., 2009). We found that tendon CD31 content in the D9 group was significantly higher than preterm^COMBINED^. There was a non‐significant decrease between D9 and D19, and it would be interesting to have an earlier and later time point in order to gain more knowledge on the postnatal development of blood vessels in tendon tissue. The data on LOX, an enzyme that plays a role in cross‐link formation and regulates fibrillogenesis (Herchenhan et al., 2015), has not been investigated before in this context. Furthermore, tendon progenitor cells exhibit high expression of SCX and TNMD, encoding two proteins important for cellular differentiation and the organization of extracellular matrix in the tendon (Liu et al., 2014; Tozer & Duprez, 2005). We find an upregulation of mRNA of these proteins in the D9 and D19 groups, while Fan et al. (2022) observed peak gene expression of *SCX*, *COL1A1* and *TNMD* in mice at postnatal day 4 followed by a subsequent decline until postnatal day 28.

### 4.4 Hormonal axis

There is evidence that the somatotrophic axis is inducible in neonates (Lewis et al., 2000), as infusion of rhGH has resulted in marked increase of hepatic IGF‐1 mRNA. Muscle IGF‐1 mRNA has also shown a significant response to rhGH, and a direct hypertrophic effect of IGF‐1 has been found in human muscle cell culture, when the treatment was administered both before and after proliferation has ceased (Velloso, 2008).

The evidence to support the same effect in response to systemic infusion of rhGH or IGF‐1 is not perfectly clear (Velloso, 2008). IGF‐1 is an inhibitor of pituitary growth hormone (GH) release in the negative feedback loop of the somatotrophic axis. There are studies that show a downregulation of IGF‐1 mRNA expression in response to IGF‐1 infusion (Frost et al., 2002; Louveau et al., 1996), even in cell cultures that were already stimulated with GH, which by itself increases IGF‐1 mRNA expression (Frost et al., 2002). Consequently, increased circulatory levels of IGF‐1 by exogenous administration, as seen in our study, might have an overall effect of downregulating the somatotrophic axis, and inhibiting the response in muscle tissue. This hypothesis would still be consistent with the effect demonstrated in cell cultures, as they more accurately serve as a model of a local tissue environment, and therefore are not directly translatable to a physiological whole organism model. It should be noted that we found no significant difference in the expression of IGF‐1 mRNA in tendon tissue between the preterm^IGF‐1^ and preterm^CON^ piglets.

### 4.5 Conclusion

The present study observed no significant effect of IGF‐1 treatment on skeletal muscle and tendon traits in preterm piglets within the first 19 postnatal days. Muscle and tendon CSA were identical in IGF‐1 treated and control preterm piglets, as were markers of tendon growth, fibrillogenesis and collagen maturation, muscle fibre type distribution, growth performance and satellite cell pool size. Further understanding of the impact of prematurity on the molecular mechanisms of growth and development in the context of all organ systems is necessary to conclude whether IGF‐1 treatment in individuals born prematurely is beneficial or not.

## AUTHOR CONTRIBUTIONS

The study was conducted at Faculty of Health and Medical Sciences, University of Copenhagen, Frederiksberg, Denmark and Institute of Sports Medicine Copenhagen, Copenhagen University Hospital, Copenhagen, Denmark. Malene Tangbjerg, Ann Damgaard, Anders Karlsen, Rene B. Svensson, Peter Schjerling, Stanislava Pankratova, Per Torp Sanild, Michael Kjaer and Abigail L. Mackey designed the study. Malene Tangbjerg, Ann Damgaard, Anders Karlsen, Rene B. Svensson and Miriam Gelabert‐Rebato conducted the experiments and/or collected the data. Malene Tangbjerg and Ann Damgaard performed the statistical analysis. Malene Tangbjerg, Ann Damgaard, Rene B. Svensson, Peter Schjerling and Abigail L. Mackey analysed and interpreted the data. Malene Tangbjerg, Ann Damgaard, Rene B. Svensson and Abigail L. Mackey wrote the manuscript. The manuscript was revised critically for important intellectual content by Anders Karlsen, Peter Schjerling, Miriam Gelabert‐Rebato, Stanislava Pankratova, Per Torp Sanild and Michael Kjaer. The final version was approved by all authors. The authors agreed to be accountable for all aspects of the work in ensuring that questions related to the accuracy or integrity of any part of the work are appropriately investigated and resolved. All persons designated as authors qualify for authorship, and all those who qualify for authorship are listed.

## CONFLICT OF INTEREST

The authors declare no conflicts of interest.

## FUNDING INFORMATION

The University of Copenhagen received financial support from Takeda Pharmaceutical Company, MA, USA and Oak Hill Bio Ltd. These funders were not involved in the study execution, collection, analysis, interpretation of data or the drafting of this article.

## Supporting information

Overview of immunofluorescence stains; mRNA primers; and statistical analyses and P‐values of muscle and tendon data.

## Data Availability

Data are available upon reasonable request to the authors.
