# Supplementary material for: Insulin‐like growth factor‐1 infusion in preterm piglets does not affect growth parameters of skeletal muscle or tendon tissue
Source: Exp Physiol. 2024 Jul 9;109(9):1529–44. doi: 10.1113/EP092010 (PMC11363143; doi:10.1113/EP092010)
Supplement: Supplementary file 1 — Overview of immunofluorescence stains; mRNA primers; and statistical analyses and P‐values of muscle and tendon data. [file EPH-109-1529-s001.docx]

**Supplementary files**

**Supplementary file 1: Overview of Immunofluorescence Stains**

| Staining Protocol | Reagent | Antibody Dilution | | Antibody Type | | Species | Company (Catalog #) |
| --- | --- | --- | --- | --- | --- | --- | --- |
| Muscle CSA, Fibre Type and Central Nuclei | Myosin Heavy Chain 2A | 1:15 | | Primary (IgG1) | | Mouse | DSHB (SC-71) |
|  | Anti-Mouse IgG Alexa Fluor 488 | 1:500 | | Secondary (IgG) | | Goat | ThermoFisher  (A-11126) |
|  | WGA  Alexa Fluor 680 |  | |  | |  | ThermoFisher (W32465) |
|  | Mounted with DAPI |  | |  | |  | ThermoFisher  (P36931) |
| Muscle Satellite Cells | Pax7 | 1:50 | | Primary (IgG1) | | Mouse | DSHB (PAX7) |
|  | Dystrophin | 1:250 | | Primary (IgG2b) | | Mouse | Sigma-Aldrich (D8168) |
|  | Anti-Mouse IgG1 Alexa Fluor 488 | 1:500 | | Secondary | | Goat | ThermoFisher  (A-21121) |
|  | Anti-Mouse IgG2b  Alexa Fluor 568 | 1:500 | | Secondary | | Goat | ThermoFisher  (A-21144) |
|  | Hoechst 33342 |  | |  | |  | ThermoFisher (H1399) |
|  | WGA Alexa Fluor 680 |  |  | |  | | ThermoFisher (W32465) |
|  | Mounted without DAPI |  |  | |  | | ThermoFisher (P36930) |

| Tendon Vascularisation | CD31 | 1:100 | Primary | Rabbit | Abcam (AB28364) |
| --- | --- | --- | --- | --- | --- |
|  | Laminin | 1:10 | Primary | Mouse | DSHB (2E8) |
|  | Anti-Mouse IgG Alexa Fluor 488 | 1:500 | Secondary | Donkey | ThermoFisher  (A-21202) |
|  | Anti-Rabbit IgG Alexa Fluor 568 | 1:500 | Secondary | Donkey | ThermoFisher  (A-10042) |
|  | Mounted with DAPI |  |  |  | ThermoFisher  (P36931) |
| Elastin Content | Elastin | 1:200 | Primary | Mouse | Abcam (AB9519) |
|  | Laminin | 1:10.000 | Primary | Rabbit | Dako (Z0097) |
|  | Anti-Mouse IgG Alexa Fluor 488 | 1:500 | Secondary | Donkey | ThermoFisher  (A-21202) |
|  | Anti-Rabbit IgG Alexa Fluor 568 | 1:500 | Secondary | Donkey | ThermoFisher  (A-10042) |
|  | Mounted with DAPI |  |  |  | ThermoFisher  (P36931) |

**Supplementary file 2: Table of mRNA primers**

Table PRIMERS:

| mRNA | Genbank ID | Sense | Antisense |
| --- | --- | --- | --- |
| Ribosomal protein lateral stalk subunit P0 (RPLP0) | NM_001098598.1 | GGAAACTCTGCATTCTCGCTTCCT | CCCGCTTGTACCCATTGATGATAG |
| Glyceraldehyd-3-phosphate dehydrogenase (GAPDH) | NM_001206359.1 | CGATGGTGAAGGTCGGAGTGAA | GTCCACTTTGCCAGAGTTAAAAGCAG |
| Cartilage oligomeric matrix protein (COMP) | XM_003123527.3 | TCCTACCGCTGGTTCCTACAGCAC | CCGCCCCGCATGGTTGTATC |
| Decorin (DCN) | NM_213920.1 | GGCTGGCAGAGCATAAGTACATCC | GTTGTATCCAGGCGGGCAGAAG |
| Collagen type I (COL1A1) | XM_021067153.1 | GGCAACAGCCGCTTCACCTAC | CGGGAGGTCTTGGTGGTTTTGT |
| Collagen type III  (COL3A1) | NM_001243297.1 | TGGTTCTCGTAAAAACCCTGCTCGT | CCATTTTGCAGCCTTGGTTAGGA |
| Insulin-like growth factor-1 (IGF-1) | NM_214256.1 | GCTGGTGGACGCTCTTCAGTTC | CGACTGCTGGAGCCGTACCC |
| Perilipin 1 (PLIN1) | NM_001038638.1 | TGTCCTTGTCTGACGCCCTGAA | AACTCGCTCTCGGGCTCCATC |
| TenascinC (TNC) | NM_214230.2 | CTGATGGGGAGATACGGGGACA | TGGGTCTCAGCTTCATCTCAGCAA |
| Tenomodulin (TNMD) | NM_001099934.1 | GCCAGGCAAGCAAGTGAGGAAG | GCCTCGACGGCAGTAGATACAACA |
| Lysyl oxidase (LOX) | NM_001206403.1 | CCTCGGGCTGCACAATTTCAC | GGCACTGATTTATCCATTGGGAGTTG |
| Matrix metallopeptidase 9 (MMP9) | NM_001038004.1 | GTGCCCTTGAACACACACGACA | AAGCGGGCCTGGCAGAAGTAAG |
| Matrix metallopeptidase 14 (MMP14) | NM_214239.3 | CCAAAAACATCAAAGTCTGGGAAGGA | TGAACTTCCAGTATTTGTTCCCCTTGT |
| Elastin (ELN) | XM_021073747.1 | AAGCTGCCCGAGGGTGACTTG | GCGCCTAGGATGCAGGGACA |
| Scleraxis (SCX) | XR_002343667.1 | GCAGCAGTGTTCTCCTCCCCAAA | GCCGCATCGGCCTCTAACTC |
| Collagen type 5 (COL5A1) | NM_001014971.1 | AGTGAATTCAAGCGCGGCAAAC | GTGATGTTCTGGTGGGCAGAGG |
| MKI67 | XM_021073741.1 | AAGCTGCCCGAGGGTGACTTG | GCGCCTAGGATGCAGGGACA |

| **Groups** | **Dependent variable** | **Statistical test** | **P-value** | **Post Hoc test** | **P-values** | |
| --- | --- | --- | --- | --- | --- | --- |
| ALL AGE GROUPS | Mean MyHC-IIa negative CSA | One Way ANOVA | **0.01** | Holm-Sidak | D9 v. Preterm COMBINED | 0.233 |
|  |  |  |  |  | **D19 v. Preterm COMBINED** | **0.022** |
|  |  |  |  |  | **D9 v. D19** | **0.012** |
| ALL AGE GROUPS | Mean MyHC-IIa CSA | One Way ANOVA | **<0.001** | Holm-Sidak | **D9 v. Preterm COMBINED** | **<0.001** |
|  |  |  |  |  | **D19 v. Preterm COMBINED** | **<0.001** |
|  |  |  |  |  | **D9 v. D19** | **0.004** |
| ALL AGE GROUPS | CSA (Weighted Mean) | One Way ANOVA | **<0.001** | Holm-Sidak | **D9 v. Preterm COMBINED** | **0.002** |
|  |  |  |  |  | **D19 v. Preterm COMBINED** | **<0.001** |
|  |  |  |  |  | **D9 v. D19** | **0.003** |
| ALL AGE GROUPS | % of MyHC-IIa neg. fibres | One Way ANOVA | **<0.001** | Holm-Sidak | D9 v. Preterm COMBINED | 0.148 |
|  |  |  |  |  | **D19 v. Preterm COMBINED** | **<0.001** |
|  |  |  |  |  | D9 v. D19 | 0.058 |
| ALL AGE GROUPS | Within-group Mean MyHC-IIa neg. CSA Compared to Mean MyHC-IIa CSA | t-tests |  |  | **Preterm CON** | **0.002** |
|  |  |  |  |  | **Preterm IGF-1** | **<0.001** |
|  |  |  |  |  | D9 | 0.472 |
|  |  |  |  |  | 19 | 0.381 |
| PRETERM | Mean MyHC-IIa neg. CSA | t-test | 0.875 |  | | |
|  |  |  |  |  |  |  |
| PRETERM | Mean MyHC-IIa CSA | t-test | 0.766 |  | | |
|  |  |  |  |  |  |  |
| PRETERM | CSA (Weighted Mean) | t-test | 0.826 |  | | |
|  |  |  |  |  |  |  |
| PRETERM | % of MyHC-IIa neg. fibres | t-test | 0.835 |  | | |
|  |  |  |  |  |  |  |

**Supplementary file 3: Statistic analyses and P-values of muscle and tendon data**

| ALL AGE GROUPS | Central nucleated fibers/100 fibers | Kruskal-Wallis (ANOVA on Ranks) | 0.139 |  | | |
| --- | --- | --- | --- | --- | --- | --- |
|  |  |  |  |  |  |  |
|  |  |  |  |  |  |  |
| ALL AGE GROUPS | Central nucleated MyHC-IIa neg. fibers/100 MyHC-IIa neg. fibers | Kruskal-Wallis (ANOVA on Ranks) | 0.234 |  | | |
|  |  |  |  |  |  |  |
|  |  |  |  |  |  |  |
| ALL AGE GROUPS | Central nucleated MyHC-IIa fibers/100 MyHC-IIa fibers | Kruskal-Wallis (ANOVA on Ranks) | **0.006** | Dunn's Metod | D9 v. Preterm COMBINED | 1 |
|  |  |  |  |  | **D19 v. Preterm COMBINED** | **0.012** |
|  |  |  |  |  | **D9 v. D19** | **0.012** |
| PRETERM | Central nucleated fibers/100 Fibers | Mann-Whitney Rank Sum Test | 0.225 |  |  | |
|  |  |  |  |  |  |  |
| PRETERM | Central nucleated MyHC-IIa neg. fibers/100 MyHC-IIa neg. fibers | Mann-Whitney Rank Sum Test | 0.532 |  |  | |
|  |  |  |  |  |  |  |
| PRETERM | Central nucleated MyHC-IIa fibers/100 MyHC-IIa fibers | Mann-Whitney Rank Sum Test | 0.305 |  |  | |
|  |  |  |  |  |  |  |
| ALL AGE GROUPS | SCs/Total Fiber Count | One Way ANOVA | 0.109 |  |  | |
|  |  |  |  |  |  |  |
| PRETERM | SCs/Total Fiber Count | t-test | 0.897 |  | | |
|  |  |  |  |  |  |  |

| **Groups** | **Dependent variable** | **Statistical test** | **P-value** | **Post Hoc test** | **P-values** | |
| --- | --- | --- | --- | --- | --- | --- |
| ALL AGE GROUPS | Tendon mRNA | One Way ANOVA |  |  | See Results, section ‘Tendon gene expression’ |  |
| ALL AGE GROUPS | CD31% | Kruskal-Wallis (ANOVA on Ranks) | **0.045** | Dunn's Method | **D9 v. Preterm COMBINED** | **0.041** |
|  |  |  |  |  | D19 v. Preterm COMBINED | 1 |
|  |  |  |  |  | D9 v. D19 | 0.214 |
| ALL AGE GROUPS | ELASTIN% | Kruskal-Wallis (ANOVA on Ranks) | **0.029** | Dunn's Method | **D9 v. Preterm COMBINED** | **0.036** |
|  |  |  |  |  | D19 v. Preterm COMBINED | 0.418 |
|  |  |  |  |  | D9 v. D19 | 1 |
| ALL AGE GROUPS | NUCLEI % | Kruskal-Wallis (ANOVA on Ranks) | 0.052 |  | | |
|  |  |  |  |  |  |  |
|  |  |  |  |  |  |  |
| ALL AGE GROUPS | LAMININ % | Kruskal-Wallis (ANOVA on Ranks) | 0.541 |  | | |
|  |  |  |  |  |  |  |
|  |  |  |  |  |  |  |
| PRETERM | CD31 % | Mann-Whitney Rank Sum Test | 0.777 |  | | |
|  |  |  |  |  |  |  |
| PRETERM | ELASTIN% | Mann-Whitney Rank Sum Test | 0.865 |  | | |
|  |  |  |  |  |  |  |
| PRETERM | NUCLEI % | Mann-Whitney Rank Sum Test | 0.061 |  | | |
|  |  |  |  |  |  |  |
| PRETERM | LAMININ % | Mann-Whitney Rank Sum Test | 0.207 |  | | |
|  |  |  |  |  |  |  |

| ALL AGE GROUPS | COLLAGEN | Kruskal-Wallis (ANOVA on Ranks) | 0.379 |  |
| --- | --- | --- | --- | --- |
|  |  |  |  |  |
|  |  |  |  |  |
| ALL AGE GROUPS | ORANGE | Kruskal-Wallis (ANOVA on Ranks) | 0.099 |  |
|  |  |  |  |  |
|  |  |  |  |  |
| ALL AGE GROUPS | VOIDS | Kruskal-Wallis (ANOVA on Ranks) | 0.265 |  |
|  |  |  |  |  |
|  |  |  |  |  |
| ALL AGE GROUPS | YELLOW | Kruskal-Wallis (ANOVA on Ranks) | 0.532 |  |
|  |  |  |  |  |
|  |  |  |  |  |
| ALL AGE GROUPS | PINK | Kruskal-Wallis (ANOVA on Ranks) | 0.738 |  |
|  |  |  |  |  |
|  |  |  |  |  |
| ALL AGE GROUPS | YELLOW+ORANGE +PINK | Kruskal-Wallis (ANOVA on Ranks) | 0.406 |  |
|  |  |  |  |  |
|  |  |  |  |  |
| PRETERM | COLLAGEN | Mann-Whitney Rank Sum Test | 0.121 |  |
|  |  |  |  |  |
| PRETERM | ORANGE | Mann-Whitney Rank Sum Test | 0.905 |  |
|  |  |  |  |  |
| PRETERM | VOIDS | Mann-Whitney Rank Sum Test | 0.551 |  |
|  |  |  |  |  |
| PRETERM | YELLOW | Mann-Whitney Rank Sum Test | 0.633 |  |
|  |  |  |  |  |
| PRETERM | PINK | Mann-Whitney Rank Sum Test | 0.17 |  |
|  |  |  |  |  |
| PRETERM | YELLOW+ORANGE +PINK | Mann-Whitney Rank Sum Test | 0.095 |  |
|  |  |  |  |  |

| **Groups** | **Dependent variable** | **Statistical test** | **P-value** | **Post Hoc test** | **P-values** |
| --- | --- | --- | --- | --- | --- |
| PRETERM | RPLP0 | t-test | 1 |  |  |
|  | GAPDH |  | 0.707 |  |  |
|  | COMP |  | 0.168 |  |  |
|  | Decorin |  | 0.683 |  |  |
|  | COL1A1 |  | 0.834 |  |  |
|  | COL3A1 |  | 0.087 |  |  |
|  | COL5A1 |  | 0.869 |  |  |
|  | IGF1 |  | 0.436 |  |  |
|  | PLIN1 |  | 0.906 |  |  |
|  | TenascinC |  | 0.11 |  |  |
|  | Tenomodulin |  | 0.746 |  |  |
|  | LOX |  | 0.082 |  |  |
|  | MMP14 |  | 0.791 |  |  |
|  | Elastin |  | 0.498 |  |  |
|  | Scleraxis |  | 0.64 |  |  |
|  | mKi67 |  | 0.984 |  |  |
